# Left atrial appendage aneurysm presenting with chronic cough

**DOI:** 10.1007/s12471-017-1021-0

**Published:** 2017-07-20

**Authors:** M. Toufan, L. Pourafkari, A. Afrasiabi, M. Sohrabi, N. D. Nader

**Affiliations:** 1 Cardiovascular Research Center, Tabriz University of Medical Sciences, Tabriz, Iran; 2 Department of Anesthesiology, University at Buffalo, New York, USA

A 32-year-old female presented with a 6-month history of worsening non-productive chronic cough and palpitations on moderate exercise. Chest X‑ray showed an increased cardiothoracic ratio with a prominent left heart border. She underwent transthoracic echocardiography in which a large cystic structure with compressive effect on the left ventricle was identified (Fig. 1a). Further evaluation by transoesophageal echocardiography proved the structure to be a large left atrial appendage aneurysm (LAAA) (7.7 × 4.4 cm) with a 1 cm entrance to the atrial cavity (Fig. 1b). The patient underwent aneurysm resection under cardiopulmonary bypass. Fig. 1c shows the resected aneurysm, which had a very thin wall. The postoperative course was uneventful.

**Fig. 1:**
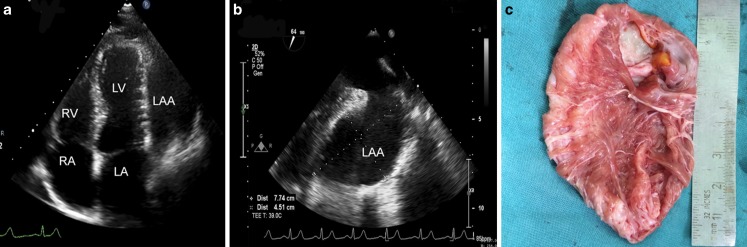
**a** Transthoracic four chamber echocardiogram showing the aneurysmal left atrial appendage with compressive effect on left ventricle, **b** Transoesophageal echocardiogram obtained at mid-oesophageal level in 60° showing the left atrial appendage aneurysm, **c** Intraoperative image showing the resected left atrial appendage aneurysm, *LA* left atrium, *LAA* left atrial appendage, *LV* left ventricle, *RA* right atrium, *RV* right ventricle

LAAA are rarely encountered and generally present with palpitations, chest pain, dyspnoea or thromboembolic events [1]. The chronic cough could have been caused by the mechanical airway compression by LAAA. Once diagnosed, surgery is warranted regardless of presence of symptoms.

## Caption Electronic Supplementary Material

Apical four-chamber trans-thoracic echocardiogram showing the large cystic structure that is compressing on the left ventricle

Modified three-chamber transthoracic echocardiogram showing the distortion of the left ventricle contour by the cystic structure

Transoesophageal echocardiogram at 60 degrees midoesophageal level showing the left atrial appendage aneurysm

Transoesophageal echocardiogram at 60 degrees midoesophageal level showing the blood flow in the left atrial appendage aneurysm
